# Statistical learning techniques applied to epidemiology: a simulated case-control comparison study with logistic regression

**DOI:** 10.1186/1471-2105-12-37

**Published:** 2011-01-27

**Authors:** John J Heine, Walker H Land, Kathleen M Egan

**Affiliations:** 1 H. Lee Moffitt Cancer Center & Research Institute, Tampa, FL, 33612, USA; 2 Binghamton University, Bioengineering Department, Binghamton, NY, USA

## Abstract

**Background:**

When investigating covariate interactions and group associations with standard regression analyses, the relationship between the response variable and exposure may be difficult to characterize. When the relationship is nonlinear, linear modeling techniques do not capture the nonlinear information content. Statistical learning (SL) techniques with kernels are capable of addressing nonlinear problems without making parametric assumptions. However, these techniques do not produce findings relevant for epidemiologic interpretations. A simulated case-control study was used to contrast the information embedding characteristics and separation boundaries produced by a specific SL technique with logistic regression (LR) modeling representing a parametric approach. The SL technique was comprised of a kernel mapping in combination with a perceptron neural network. Because the LR model has an important epidemiologic interpretation, the SL method was modified to produce the analogous interpretation and generate odds ratios for comparison.

**Results:**

The SL approach is capable of generating odds ratios for main effects and risk factor interactions that better capture nonlinear relationships between exposure variables and outcome in comparison with LR.

**Conclusions:**

The integration of SL methods in epidemiology may improve both the understanding and interpretation of complex exposure/disease relationships.

## Background

The objectives of this work are to 1) demonstrate the benefits of applying statistical learning (SL) concepts to epidemiologic type problems using simulated data when nonlinearities are present, and 2) adapt the SL approach to produce findings relevant for epidemiologic interpretation. Statistical learning effectively describes statistical estimation with small samples [1]. The approach does not rely on prior knowledge of the mathematical form of the exposure/disease relationship, an assumption in parametric modeling. A more detailed account of SL theory is provided elsewhere [1,2].

A comparison of a kernel based SL technique with logistic regression (LR) modeling was developed using simulated case-control datasets with a focus on the separation boundary and information embedding characteristics of both approaches. Illustrations were developed to demonstrate how the kernel mapping addresses the nonlinearity without user imposition. Without loss of generality, a low-dimensional problem was used to demonstrate the central themes because the separation boundaries can be observed graphically, which is not the case for higher-dimensional problems. The comparison with LR serves three purposes. First, although LR modeling is widely used for epidemiologic applications, its separation boundary represents a latent characteristic that is often not considered directly. Secondly, the information embedding characteristic of LR is representative of parametric approaches. The possible benefits derived from applying a kernel based technique come with a tradeoff in comparison with parametric modeling of requiring training data for prospective analyses. Thirdly, the LR model has an important epidemiologic interpretation. Therefore, the SL approach was modified to conform to the LR model interpretation.

Epidemiologic research makes frequent use of LR modeling for determining relationships between covariates and group associations when the outcome is binary. We will refer to the group association as the binary disease status and refer to covariates as *risk factors or exposures*. Logistic regression has many attractive attributes in this setting. The model coefficients are related to odds ratios (ORs) by exponentiation, which convey relevant exposure/disease association relationships. The LR model is a generalized linear model [3,4]. Various methods have been investigated to generalize such relationships in epidemiologic research. Neural network (NNs) have been used in studies of immunodeficiency viral infection [5] and liver disease [6,7]. Other researchers modified the LR model to include non-parametric functions to study colon cancer [8]. Generalized models have also been used in various capacities to model lung function change [9], blood pressure [10], alcohol consumption [11], and heart disease [12].

We will consider a dataset assembled from a case-control study in which each observation contains information on the binary disease status and a set of associated exposures. These exposures can be assembled into one vector, **x**, for each observation, which we label as the *input*. Hypothetically, there is some relation f(**x**) that describes the separation boundary between the case and control groups to some specified degree, where the group status is the *output*. Otherwise, **x **would not show association with disease. In a multivariate setting, the separation, or decision boundary, is a hyper-surface that reduces to a hyper-plane when **x **and the disease status bear a linear relation. Error in predicting group status may occur from a number of sources including inferior model specification, complicated relationships between the exposure distributions and group status, random error, non-random measurement error, or some combination of these influences. In practice, decision models rarely, if ever, produce perfect class-separation when making predictions.

We will consider a model encompassing two-exposures for each observation [i.e., a two-dimensional input vector **x **= (x_1_, x_2_) for each observation] in which the solutions and covariate relationships can be viewed in a two-dimensional plane by design. For a linearly separable two-dimensional problem, the input/output separation boundary is a straight line. When this problem is nonlinear separable, the input/output separation boundary is a curve (one dimensional) of some form. In practice, f(**x**) is rarely known. Interaction terms (or other functional forms) can be introduced within the LR model to capture the attributes of f(**x**), which are discernable graphically in a two-dimensional problem. However, in higher dimensional problems, it may not be clear whether the modified LR model provides a correct fit of the data. The two-dimensional problem demonstrated herein is used for illustrative purposes though it is representative of higher dimensional problems that are difficult or impossible to observe and model by intuition.

Odds ratios and the area under the receiver operator characteristic (ROC) curve, designated as Az, are used for comparing group characteristics for different purposes. When model predictive capability is important, Az is often used as the measure of separation in two-class problems [13-15]. In epidemiologic research, ORs are used to gauge the magnitude of association between exposure and outcome. In contrast with the LR model, the SL approach does not produce a data representation that has a useful epidemiologic interpretation. Therefore, we present non-parametric probabilistic methods that can be used for converting SL outputs to more readily interpretable ORs. We also calculated the Az quantity for each model used in the comparison analysis because it is measure of how well the models fit the data. The relationship between ORs and ROC analysis has been previously described [16].

In this report, a SL technique comprised of the kernel mapping in combination with a perceptron NN [17] was compared with the LR modeling. Kernel mappings are used to capture the non-linear relationship between the input/output without prior knowledge of the form of f(**x**). We simulated data from a case-control study, which is a study-design employed in our ongoing epidemiologic research [18,19]. The goals of this ongoing research are analogous to those of Phase I or Phase II clinical studies wherein the objective is to determine whether certain exposures or measurements are more (or less) likely to be associated with a targeted disorder [20], where the disorder in our work is breast cancer. There is no explicit intent to make predictions at the population level at this time, though our methods could be adapted for this purpose in the future.

## Methods

**Figure 1:**
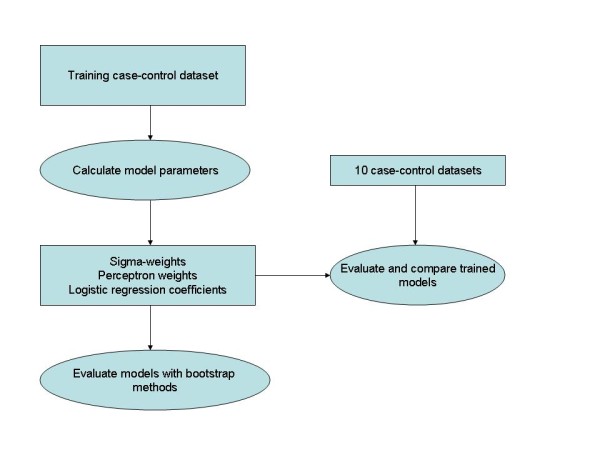
**Training and evaluation scheme**. This figure shows the analysis sequence. One simulated training dataset was used to estimate the parameters for all models and perform an initial evaluation with the fitted models. Ten independent simulated datasets were then used to evaluate the fitted models to eliminate training bias.

An overview of the multiple steps used for this analysis is shown in Figure 1. Briefly, we simulated one training dataset that was used exclusively to determine all of the model parameters for both the LR and SL approaches and perform an initial evaluation. We then evaluated the fitted models with multiple independent simulated datasets (validation datasets) to estimate the variation in the model performance.

### Simulated Case-Control Study

A simulated case-control dataset was generated with m = 200 observations in each of the case and control groups, which is a relatively small sample size by design. Both random variables (rvs) and their respective realizations are denoted by lower case letters, and vectors are similarly labeled with bold letters. To avoid using transpose notation, all vectors are defined as row vectors. Each observation (simulated study subject) has two risk factors denoted by x_1 _and x_2 _expressed as a vector **x **= (x_1_, x_2_). We used an *activation *function to randomly generate the disease status defined as

(1) $$\text{g}({\text{x}}_{1})=\frac{1}{{\text{c}}_{0}}\times \left(\frac{{\text{x}}_{1}^{2}}{{\text{x}}_{1}^{2}+{\left(1-{\text{x}}_{1}\right)}^{2}}+\exp[-{\left({\text{a}}_{0}{\text{x}}_{1}-{\text{m}}_{0}\right)}^{2}]\right),$$

where a_0_, c_0_, and m_0 _are adjustable constants. This expression provides a flexible nonlinear boundary. The left term within the brackets is a sigmoidal function constructed from a parabola [21] and the right term gives a scalable spatially adjustable bulge. The disease status is dependent upon a given observation's **x **composition by this relation: g(x_1_) > x_2_. When this condition is met, the given observation is placed in the case group with its known risk factor vector **x **= (x_1_, x_2_). Otherwise, the observation is designated as control group member with the same vector **x **= (x_1_, x_2_). In this example, g(x_1_) assumes the position of the unknown function f(**x**) discussed above. Equation (1) in combination with the defined case-control designation rule is an rv transformation for x_1 _that creates a nonlinear separation boundary stochastically.

#### Simulated case-control datasets

We generated one case-control dataset for training (model fitting to determine all parameters) and ten additional validation datasets for evaluation purposes using the following prescription (11 datasets in total). To generate a given case-control dataset, 20,000 observations of (x_1_, x_2_) were generated randomly and processed with g(x_1_), which created the case-control designation. The first m observations from each group were used to form a given case-control dataset resulting in 2m observations with equal numbers of cases and controls (m controls and m cases). The x_1 _observations were uniformly distributed rvs with unit variance. The x_2 _observations were generated by adding x_1 _to a normally distributed rv, designated as z_1_, with unit variance and mean = 5 giving x_2_= (x_1_+z_1_)/10. The empirical linear correlation between x_1 _and x_2 _after the g(x_1_) processing was estimated as R = 0.25.

### Decision Models

The model construction, training methods, evaluation, and separation boundary analysis are described below in detail. Simulated case-control datasets were modeled with two LR models and three SL variants. Training (in which we estimate the model parameters) and model evaluations were performed with independent datasets to eliminate fitting bias in the comparison analysis. In the model comparison analysis, both predictive capability (i.e., Az) and ORs were compared. The training and evaluation sequences are shown in Figure 1.

#### Statistical learning overview

First, the kernel mapping was applied to the input vectors. The kernel-transformed data was then processed by a perceptron [17] using an algorithm described previously [22]. The perceptron can be used to solve a system of linear equations where each equation is of the form y = **r·w**+b. In this expression, **w **is arbitrary weight vector, **r **is an arbitrary risk factor vector similar to **x **above, b is a constant, and y is a two-class binary variable representing the disease/no-disease status (i.e., y = 1, or y = -1). Hereafter, we refer to the kernel mapping and perceptron combination as the SL approach. The perceptron weight determination will converge when the problem is well approximated a linear-separable.

#### Kernel mapping

We will use a kernel mapping to express the input such that it is suitable for the perceptron processing. Under general circumstances, the researcher will find it difficult, if not impossible, to specify the mapping function that provides for a linear separation boundary. The kernel operates on the risk factor vectors and eliminates the need to determine the general mapping function denoted by ϕ(**x**). We use ϕ(**x**) for the mapping function, which is the transformation that renders the input/output relationship linear if chosen properly, because it conforms with the standard notation used in SL developments. As defined above, each observation has an associated risk factor vector, where **x**_j _= (x_1j_, x_2j_) designates the j^th ^training sample's vector, and **x **= (x_1_, x_2_) is used specifically to designate an arbitrary prospective observation's vector (not a training sample). Reproducing Kernel Hilbert Space theory states that a suitable kernel can be defined as the inner product of the mapping functions [23] expressed as

(2) $$\text{k}(\text{x},{\text{x}}_{\text{j}})=\langle \phi (\text{x}),\phi ({\text{x}}_{\text{j}})\rangle ,$$

where **x **is a prospective observation (random) vector, with the same dimensionality as **x**_j_, and 〈·,·〉 is the inner product operation. The challenge changes from finding the mapping function to finding a valid kernel (there are many) as described previously [24]. The right side of Eq. (2) allows for the use of the left side without knowing the form of the right side. To define the specific kernel used here, we first define the distance measure between the vectors **x **and **x**_j _given by

(3) $$\text{D}(\text{x},{\text{x}}_{\text{j}})=\sqrt{\frac{{\text{s}}_{1}{\left({\text{x}}_{1}-{\text{x}}_{1\text{j}}\right)}^{2}}{\sigma_{1}^{2}}+\frac{{\text{s}}_{2}{\left({\text{x}}_{2}-{\text{x}}_{2\text{j}}\right)}^{2}}{\sigma_{2}^{2}}}.$$

The extension to higher dimensional vectors follows the same form by extending the sum within the radical to include more component terms. Each vector component difference has its own sigma-weight (σ_1 _and σ_2_) that was determined with training methods discussed below. These sigma-weights must be estimated properly because they impact the decision performance. We used three variations of Eq. (3). The s_1 _and s_2 _are for identifications purposes in this report only. Equation (3) was used with both component terms (s_1 _= s_2 _=1) as above and with the individual component differences in isolation with (s_1 _= 1, s_2 _= 0) when the focus was on x_1 _and (s_1 _= 0, s_2 _= 1) when the focus was on x_2_. The kernel is then defined as

(4) $$\text{k}(\text{x},{\text{x}}_{\text{j}})={\text{c}}_{\text{x}}\times \exp[-\text{D}(\text{x},{\text{x}}_{\text{j}})],$$

where c_**x **_is a normalization constant. Equation (4) with Eq. (3) is from a class of universal kernels [25]. The kernel operation represents both a mapping of the input vectors [23] and also forms the basis for estimating probability density functions [26,27].

To determine the parameters for the SL approach, we used each individual training observation as a substitute for the prospective observation by cycling through the kernel processing. More specifically, each **x**_j _training sample is processed with every other **x**_i _training sample using Eqs. (3-4) to determine both the sigma-weights and the perceptron weight vector (i.e., **x **takes on all **x**_i _for i = 0 through 2m). The i^th ^row of **K **results from the kernel operation of the i^th ^sample with each of the other 2m samples (including itself) indexed by j = 1 through 2m. The resulting kernel elements form 2m × 2m matrix, **K**, with elements k(**x**_i_, **x**_j_) = k_ij_. A given row in the **K **matrix can be considered as new feature set (or row vector) for the respective observation (patient), which is the dimensionality expansion characteristic of the SL approach. The decision rule using the trained model (determined sigma-weights and perceprton weights) to make prospective predictions on the observation **x **is given by

(5) y=ϕ(x)⋅w+b,

where y is the estimate of the binary disease status, b is an arbitrary (bias) constant, and **w **is generic weight vector. Expanding **w **in terms of the mapping function gives

(6) $$\text{w}=\sum_{\text{j}=1}^{2\text{m}}\alpha_{\text{j}}\phi ({\text{x}}_{\text{j}}).$$

Using Eq. (5) in Eq. (6) and performing the inner product gives

(7) $$\text{y}=\langle \phi (\text{x}),\sum_{\text{j}=1}^{2\text{m}}\alpha_{\text{j}}\phi ({\text{x}}_{\text{j}})\rangle +\text{b}=\sum_{\text{j}=1}^{2\text{m}}\alpha_{\text{j}}\text{k}(\text{x},{\text{x}}_{\text{j}})+\text{b},$$

which follows from the kernel inner product relation [23]. Equation (7) allows for the use of the kernel rather than the mapping function. For training, we let **x **= **x**_i _in Eq. (7) giving

(8) $${\text{y}}_{\text{i}}=\sum_{\text{j}=1}^{2\text{m}}\alpha_{\text{j}}\text{k}({\text{x}}_{\text{i}},{\text{x}}_{\text{j}})+\text{b},$$

where α_j _are the components of the new weight vector **α**. The components of **α **are the preceptron weights that were determined with the training dataset using this linear combination to predict the i^th ^training observation's known case-control status designated by y_i_.

#### Perceptron processing

We employed bootstrap methods [28] with the perceptron algorithm during the training analysis to estimate **α **in Eq. (8). In the perceptron algorithm used here, the bias term, b, is not affected by the inputs [the kernel elements in Eq. (8)] but is an externally applied value (b = 1), left unchanged during the determination of the weight vector that fixes the position of the separation boundary (but does not affect the boundary orientation). When processing a prospective sample from a given validation dataset, the prospective observation's vector, **x**, is processed with the case-control training dataset consisting of 2m known risk factor vectors. The prospective observation's estimated output score, y_est_, was generated using the Eq. (8) relationship from above

(9) $${\text{y}}_{\text{est}}=\sum_{\text{j}=1}^{2\text{m}}\alpha_{\text{j}}\text{k}(\text{x},{\text{x}}_{\text{j}})+\text{b}$$

with the previously determined **α **and b. Equation (9) demonstrates the information embedding characteristic of the kernel operation and illustrates how the mapping captures the underlying probability densities. A given kernel element (elements of **K**) can be interpreted as either 1) similarity measure between the prospective observation's vector **x **with the j^th ^training sample's vector **x**_j_, or 2) as one element of a multivariate kernel probability density estimation for **x**. Each new score (for the prospective **x**) is determined by making comparisons with the entire training set.

Each of the 2m validation observation scores for a given dataset (one of 10 datasets) was generated with the above equation by letting their risk factor vectors take the position of **x**. The dimensionality of the problem was fixed by the training methods. The number of observations in a given validation dataset is irrelevant for the mechanics of the processing. In addition to using both risk factors simultaneously, the perceptron was also trained using x_1 _and x_2 _separately with the same procedure without regenerating the sigma-weights, which created two additional SL variants used in the comparison. To standardize the associations for the three SL models, the y_est _scores derived from Eq. (9) for a given model output were treated as single unit (both cases and control scores) and linearly mapped between [0-1]; we labeled these normalized output scores as z.

#### Logistic Regression

The LR model is expressed as

(10) $$\text{P}\text{r}(\text{c}\text{l}\text{a}\text{s}\text{s}=1|\text{x})=\text{p}(\text{x})=\frac{\text{e}\text{x}\text{p}(\beta_{0}+\beta_{1}{\text{x}}_{1}+\beta_{2}{\text{x}}_{2}+\beta_{3}{\text{x}}_{1}{\text{x}}_{2})}{1+\text{e}\text{x}\text{p}(\beta_{0}+\beta_{1}{\text{x}}_{1}+\beta_{2}{\text{x}}_{2}+\beta_{3}{\text{x}}_{1}{\text{x}}_{2})},$$

where Pr indicates probability. This model was used with x_1 _and x_2 _without interaction (referred to as the standard model with β_3 _= 0) and with x_1 _× x_2 _interaction (referred to as the interaction model). The respective parameter vectors (β_0_, β_1_, β_2_) and (β_0_, β_1_, β_2_, β_3_) for each model were determined with the training dataset. We note, this model embeds information in the coefficients (on the order of the dimensionality) regardless of the number of observations on hand and is representative of parametric approaches.

#### Training and evaluation methods

Both the SL approach and LR model required training to estimate the various parameters. These models were trained with the same training dataset consisting of 2m observations. Figure 1 shows the training and evaluation flow schematic. The LR models were fitted with SAS (SAS Institute, NC) software. The SL approach required more involved training with bootstrap re-sampling [28]. Because the sigma-weights impact the performance of the perceptron output, the perceptron training was embedded within the sigma- weight estimation. Perceptron weights were determined by drawing row vectors from the **K **(training) matrix at random with replacement. The Az was used as a guide for convergence. Because there are only two sigma-weights, a constrained search was used by varying both weights over a range of values. For each sigma-weight combination, the perceptron weights were determined, and the Az value was estimated resulting in an experimental set of values: {σ_1i_, σ_2i_, Az_i_} for the i^th ^combination. The sigma-weights were determined by the position of the maximum Az value (Az_max_): σ_1 _= σ_1i _and σ_2 _= σ_2i _where Az_i _= Az_max_. Once the sigma-weights were established, the perceptron weights were regenerated (fine-tuned) by incrementally increasing the Az convergence criterion using a feedback loop. The perceptron weights that gave the highest Az before non-convergence were used in the validation processing along with {σ_1_, σ_2_}. When using x_1 _and x_2 _individually [s_1 _= 0 or s_2 _= 0 in Eq. (3)], we retrained the perceptron with the same Az criterion using the respective sigma-weights (determined above). In sum, the sigma-weight pair in combination with the perceptron weights that gave the highest Az for given SL variant were used in the model evaluation comparison.

The training dataset was used to evaluate the fitted models initially by generating 10 repetitions of 150 bootstrap datasets [28]. Each bootstrap dataset was processed by each of the models. For a given repetition, the distribution mean (Az_150_) and standard deviation (σ_150_) were calculated for each model. Averages of the Az_150 _and σ_150 _quantities were used to estimate the respective average performances and standard errors (SEs). For independent evaluation, 10 additional datasets were processed by each fitted model to estimate the average performance and SEs.

#### Separation boundary analysis

To compare the specific separation boundaries produced by the various models, it was necessary to apply a threshold to each model's output and estimate its performance. For consistency and to avoid user imposition, the same method was used to set the threshold for each model. In two class prediction problems (disease/no disease) used to assign class status, an operating point (decision threshold) must be selected from the model output, often derived from the ROC curve. This operating point represents a tradeoff between making two errors [13,14]. These are 1) the error of classifying cases as controls, defined in summary as the false negative fraction (FN), which is equivalent to 1-sensativty, where the sensitivity is the correctly identified proportion of cases, which is often referred to as the true positive fraction (TP), and 2) the error of classifying controls as cases denoted as the false positive fraction (FP) in summary. Plotting the ordered pairs, (FP, TP), for each threshold, which is a latent variable, approximates the continuous ROC curve. Choosing a threshold fixes the separation boundary. For the LR model, all samples with p(**x**) scores ≥ p_t _were classified as case group members, otherwise they were classified as control group members, where p_t _is a fixed threshold. To determine the separation boundaries, the operating point for a given model was selected by choosing the sensitivity equivalent to its Az value. Because the FP variable is defined over this range [0-1], the Az value may also be interpreted as the model's mean (average) sensitivity (i.e., the value of the area under the ROC curve is also the mean value of the ROC function). For an arbitrary threshold value, p_t_, the separation boundary for the standard LR model was found by solving Eq. (10) for x_2_, giving

(11) x2=−(τ+0β0)β2−β1β2x1

with $\tau_{0}=\text{ln}(\frac{1-{\text{p}}_{\text{t}}}{{\text{p}}_{\text{t}}})$, which is a linear boundary. Including the LR interaction term gives

(12) x2=−τ+0β0+β1x1β2+β3x1,

We will find the value of p_t _that gives a sensitivity equivalent to the Az (or the mean sensitivity) for the respective LR models to determine the separating boundaries and estimate the corresponding FP for comparison purposes. The same approach was applied to the SL output. This method used to set the thresholds eliminated user input because there are an unlimited number of thresholds to choose from, each representing a different tradeoff as described above. Our objective is to show the form of the various separation boundaries, therefore the method used to set the threshold is not important to the central demonstration.

### Odds Ratio Transformation

The SL technique output [the perceptron output defined in Eq. (9)] was modified to conform to the LR model interpretation and generate ORs. Specifically, we estimated the empirical conditional probability function p_r _= Pr(class = 1|z) as the reference, where z is the SL method normalized output score We then estimated p_1 _= Pr(class = 1|z+∆z) in the same manner, where ∆z is in positive increment in the respective z score. The ORs were calculated using this definition

(13) $$\text{OR}=\frac{{\text{p}}_{1}}{1-{\text{p}}_{1}}\times \frac{1-{\text{p}}_{\text{r}}}{{\text{p}}_{\text{r}}}.$$

Equation (13) can be applied by using all of the risk factors in the model or any subset. When using more than one risk factor, it can be considered as multivariate OR. In the Eq. (13) representation, p_r _has the analogous interpretation as the LR model in Eq. (10), although it was derived numerically. Equation (13) was generated for each of the SL variants for one of the evaluation datasets. We note that using Eq. (13) with these specific definitions for p_r _and p_1 _parallels the development used to derive the interpretation for the LR model coefficients for continuous independent variables [29].

The components (p_1 _and p_r_) in Eq. (13) were constructed as approximations for continuous functions using non-parametric techniques. To estimate p_1 _and p_r_, first the histograms of normalized output scores for the m cases and m controls were analyzed separately. A kernel density estimation technique [27] was used to generate the empirical probability densities from the output score histograms using a Gaussian kernel. The kernel density technique is a non-parametric method used to estimate the underlying probability density function given samples drawn from a given population without assumption that generalizes the respective histogram (similar to the kernel mapping). This is a particularly useful technique when the dataset is sample-limited with missing bins in the histogram because it is essentially a sifting mechanism that can eliminate discontinuities. The estimated densities for the cases and controls are denoted by h_1 _and h_0_, respectively, giving p_r _= h_1_/(h_1_+h_0_), which is a function of z. The p_1 _function was estimated similarly by shifting p_r _by ∆z.

## Results

### Model Training

Model parameters were determined and each model was assessed with the training dataset. The coefficients for the standard LR model using x_1_, and x_2 _simultaneously without interaction and with x_1 _× x_2 _interaction were: (β_0_, β_1_, β_2_) = (-7.251, -1.743, 14.33) and (β_0_, β_1_, β_2_, β_3 _) = (-13.73, 9.66, 26.03, -20.12), respectively. These coefficients are presented as log(ORs) [i.e, ln(OR)] per unit increase in the respective variables. These large values are due to the unit increase because both x_1 _and x_2 _span less than one unit. For the standard model, x_1 _provides a shielding effect with respect to the disease status (e.g., the coefficient remains negative, implying an inverse association of the factor with disease status), whereas x_2 _shows a relatively stronger positive magnitude of association in comparison with x_1_. In contrast, in the interaction model, the x_1 _and x_2 _terms both show a positive association with the outcome while the interaction term has a negative coefficient. For this initial Az assessment, averages, standard deviations, and SEs derived with bootstrap methods [28] are given in Table 1. The Az quantities for the training x_1 _and x_2 _sample distributions were also generated for comparison purposes; these Az quantities were estimated by comparing the respective distributions without model-processing. The sigma-weight pair in combination with the perceptron weights that gave the highest Az were used in the comparison evaluation: (σ_1_, σ_2 _) = (3.88, 2.47). The trained model Az findings are given in Table 1 for the three SL models.

**Table 1:** Training area under the receiver operator characteristic curve quantities

Method	Az	σ	SE
LR	0.791	0.028	0.008

LR_int_	0.814	0.027	0.008

k	0.958	0.013	0.004

k_x1_	0.867	0.023	0.007

k_x2_	0.728	0.031	0.009

x_1_	0.490	0.035	0.011

x_2_	0.772	0.029	0.009

This table gives the area under the receiver operator characteristic curve (Az) quantities derived from the training dataset for the standard logistic regression model with x_1 _and x_2 _(LR), the logistic regression model with x_1 _and x_2 _with x_1 _× x_2 _interaction (LR_int_), the statistical learning (SL) techniques using a kernel mapping with x_1 _and x_2 _simultaneously (k), and partial SL-kernel models using x_1 _(k_x1_) and x_2 _(k_x2_) individually. This also gives the Az quantities for the x_1 _and x_2 _case-control training distribution samples estimated without using model processing. Az and σ are the respective means and standard deviations summarized from the bootstrap trials. SE is the standard error in Az.

### Model Evaluation

**Figure 2:**
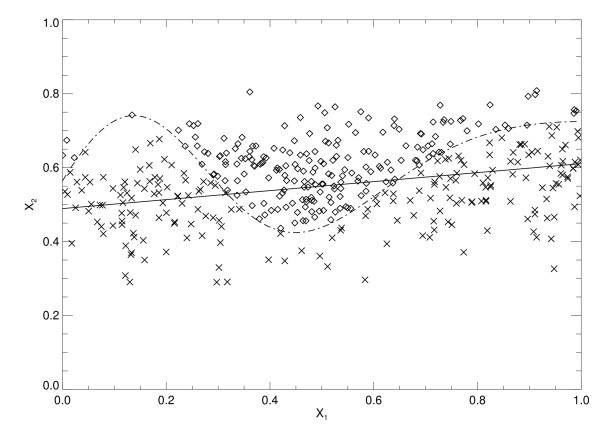
**The x**_**1**_**-x**_**2 **_**scatter plot and logistic regression boundary**. This figure shows the two risk factor scatter plot for cases (diamonds) and controls (multiplication signs). Each point represents a given sample's (x_1_, x_2_) risk vector plotted in component form. The solid line is the standard logistic regression (no-covariate interaction) model linear separation boundary for a fixed threshold and the curved dashed line is the Eq. (1) (ideal) separation boundary. The sensitivity = 0.78 and false positive fraction = 0.42

**Figure 3:**
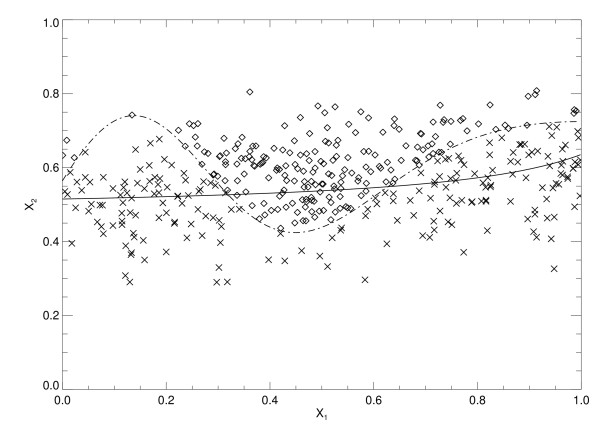
**The x**_**1**_**-x**_**2 **_**scatter plot and the logistic regression with interaction boundary**. This figure shows the two risk factor scatter plot for the cases (diamonds) and controls (multiplication signs) for the LR model with x_1 _× x_2 _interaction. Each point represents a given sample's (x_1_, x_2_) risk vector plotted in component. The solid line is the LR model separation boundary (solid) and the curved dashed line is the Eq. (1) (ideal) separation boundary. In comparison with Figure 2, there is a slight curvature in the boundary on the right side. The sensitivity = 0.80 and false positive fraction = 0.40

**Figure 4:**
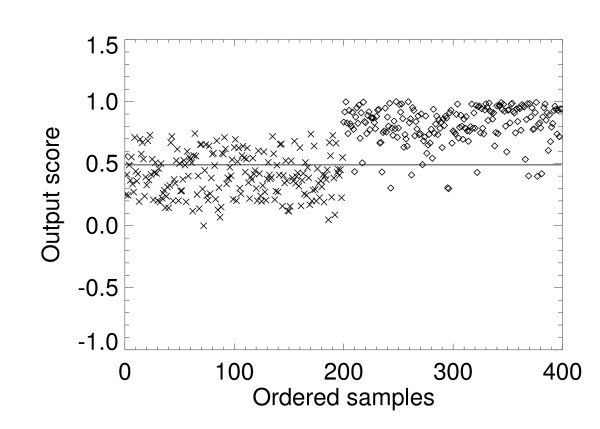
**Statistical learning (SL) output and boundary**. This figure shows the SL output separation boundary. Ordered samples are plotted along the horizontal-axis with the 200 control observations plotted first (multiplication signs on the left side) followed by the 200 case observations (diamonds on the right side). The SL output normalized z-scores for each sample are plotted on the vertical-axis. The separation boundary that gave 0.95 sensitivity is z = 0.49 (solid line) with a false positive fraction = 0.33.

The two trained LR models and the three trained SL variants were used to process the 10 validation case-control datasets (Figure 1). Summarized Az findings for all model outputs are listed in Table 2, which mirror those in Table 1. The SL approach provided the best performance. The predictive capacity of the LR model is captured in the x_2 _term by noting its coefficient. The LR model gained marginal predictive capacity by adding the interaction term as indicated by the increased Az value. In contrast, the univariate SL variants show that x_1 _in isolation contains considerable information content in comparison with x_2_. Figure 2 shows the linear separation boundary for the standard LR model plotted with the case-control data points. The solid line is the LR separation boundary derived from Eq. (11) with Az ≈ 0.78, which gave FP ≈ 0. 42 with p_t _≈ 0.42. The other curve (dashed line) in Figure 2 represents the ideal boundary that was derived with Eq. (1). Figure 3 shows the separation boundary for the LR interaction model (same format) derived from Eq. (12) with Az ≈ 0.80, which gave FP ≈ 0.40 with p_t _≈ 0.41. Figure 4 shows the SL plot derived with Az ≈ 0.95, which gave FP ≈ 0.33 with z ≈ 0.49 (the solid line separation boundary). In this plot, samples were ordered along the horizontal axis according to the observation index. The first 200 points correspond to controls and the next 200 points correspond to the cases. The respective normalized output scores are plotted on the vertical axis with the control scores denoted by multiplication signs and the case scores by diamonds. These examples illustrate the information embedding characteristics of the kernel mapping.

**Table 2:** Evaluation area under the receiver operator characteristic curve quantities

Method	Az	σ	SE
LR	0.781	0.029	0.009

LR_int_	0.798	0.029	0.009

k	0.947	0.015	0.004

k_x1_	0.852	0.018	0.005

k_x2_	0.734	0.023	0.008

This table gives the area under the receiver operator characteristic curve (Az) quantities for the standard logistic regression model using x_1 _and x_2 _(LR), the logistic regression model using x_1 _and x_2 _with x_1 _× x_2 _interaction (LR_int_), the statistical learning (SL) model using a kernel mapping with x_1 _and x_2 _simultaneously (k), and partial SL-kernel models using x_1 _(k_x1_) and x_2 _(k_2_) individually. Az, and σ are the respective means and standard deviations derived from processing the 10 validation datasets with the trained models. SE is the standard error in Az

Once the model parameters were determined for the LR models, the functional form of their separation boundaries were fixed. For example, changing the thresholds for either of the LR models will shift the boundaries (Figure 2 and Figure 3) and provide different decision performance (i.e., different sensitivity and FP) but will not alter the boundary forms. The boundary in Figure 4 illustrates that the kernel mapping transformed the input/output relation from the separation boundary shown in Figure 2 or Figure 3 to the separation shown in Figure 4.

### Odds Ratio Transformation

**Figure 5:**
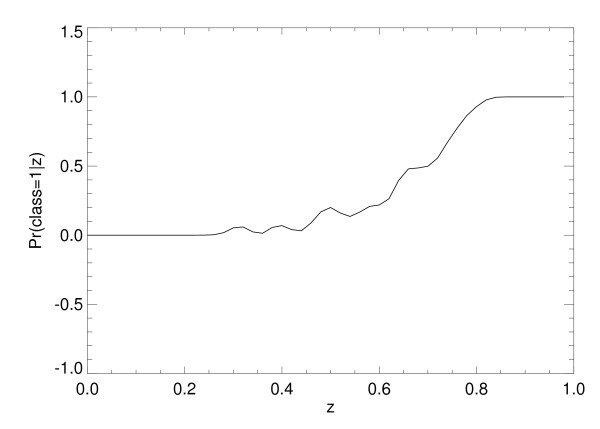
**Empirical conditional probability function estimation**. This figure demonstrates the numerical estimate of Pr(class = 1|z), where z is the statistical learning method output score using both risk factors and Pr denotes probability. The predictive capacity of the SL method is indicated by the rapid approach to Pr = 1 with increasing z (z ≈ 0.82).

**Figure 6:**
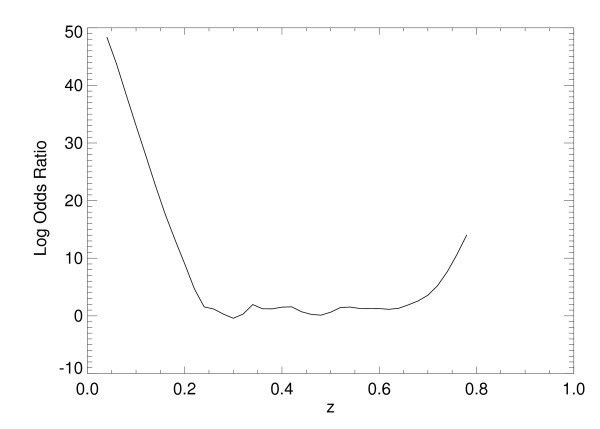
**Multivariate logarithm of the odds ratio for the two-risk factor statistical learning method output**. This figure demonstrates the log (odds ratio) [i.e., ln(odds ratio)] plot derived from the two-risk factor statistical learning method output, z, using the formulism illustrated in Figure 5 [s_1 _= 1 and s_2 _= 1 in Eq. (3)].

**Figure 7:**
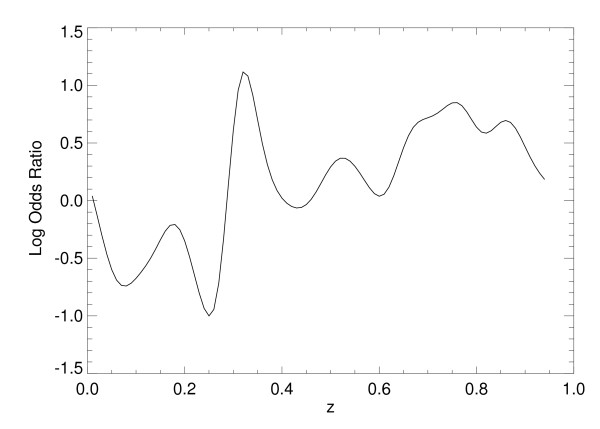
**Logarithm of the odds ratio for the statistical learning method output for the first risk factor**. This figure demonstrates the log (odds ratio) [i.e., ln(odds ratio)] plot derived from the statistical learning method output, z, using x_1 _[s_1 _= 1 and s_2 _= 0 in Eq. (3)].

**Figure 8:**
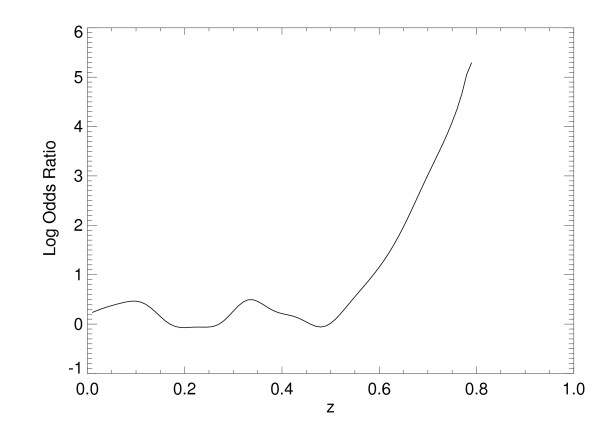
**Logarithm of the odds ratio for the statistical learning method output for the second risk factor**. This figure demonstrates the log (odds ratio) [i.e., ln(odds ratio)] plot derived from the statistical learning model output, z, using x_2. _[s_1 _= 0 and s_2 _= 1 in Eq. (3)].

Odds ratios were calculated using x_1 _and x_2 _simultaneously, as well as individually, by applying Eq. (13) to each of the SL model's normalized output scores. For SL approach with both variables, the numerical estimate of p_r _= Pr(class = 1|z) is shown in Figure 5 [same interpretation as Eq. (10)]. The ORs were then derived by letting p_1 _= Pr(class = 1|z+∆z) with ∆z = 0.10 (output-score increment units). The corresponding *continuous *log(OR) plot is shown in Figure 6, which can be considered as a multivariate OR showing the influence of both factors simultaneously. Similarly, the log(OR) plots for x_1 _and x_2_, individually, are shown in Figure 7 and Figure 8, respectively. In practice, the ORs can be rescaled. Because the problem was simulated, rescaling has little relevance. The focus of the analysis is the OR nonlinearity. These plots show the functional dependence of the ORs in comparison with the LR coefficients that are constants. When the log(ORs) derived from the SL outputs are constant, the Eq. (13) relations would approximate constant valued functions similar to the LR model coefficients, which are essentially average effects under the linear assumption.

## Discussion

A two-dimensional problem was simulated to illustrate some advantages of applying SL techniques to epidemiological type datasets. Comparisons of the Az quantities among the various models (Table 2) demonstrates the capacity of the SL approach when addressing nonlinear problems in contrast with the LR results. The SL output scores were transformed into ORs using a kernel density estimation technique. This transformation provided the essential link between the SL output and the epidemiologic interpretation for both the multivariate OR relation, which is the combined disease/risk factor association for both (all) the covariates simultaneously including their interactions, as well as the individual risk factor associations. As demonstrated, the ORs exhibit (see figures 6-8) a nonlinear functional dependence with respect to the output score. When the input/output relationship is nonlinear, the LR coefficient does not describe the association properly due to the LR model linear separation boundary. We note that the LR output could be manipulated in the same fashion, but the relationship would not capture the correct interaction because of the linear model form.

Other researchers incorporated kernel density estimations in epidemiologic research for different applications [30-32]. Similar kernel density estimations techniques were used earlier to derive relative risks [31]. Duh et al [6] provided an epidemiologic interpretation of the NN weights when using an LR type activation function. In contrast with this related work using kernel density estimations, we applied the kernel density estimation to the SL model output after the kernel mapping. This approach used the decision model outputs as new risk factor quantities that captured the inherent nonlinearities.

The kernel mapping expands the dimensionality of the problem and uses the entire training dataset for prospective analysis. This expansion enables the SL system to learn the input/output relationship, which is captured in the kernel elements and the perceptron weight vector. Each kernel element in the Eq. (9) linear combination represents a similarity measure between the respective training sample and the prospective observation. This is in contrast with parametric modeling techniques that use relatively few model coefficients to summarize the training dataset attributes. The ability of the SL approach to learn the input pattern in exemplified by the Az result for x_1 _when processed in isolation. The relatively large Az value resulting from the SL technique when including both exposure variables indicates the kernel mapping captured the nonlinear information content and transformed the original representation to a nearly linear separable representation. Generally, SL methods require more involved training than that of parametric modeling, an inevitable trade-off required to capture the nonlinearity. For higher-dimensional problems more sophisticated optimization techniques are required, such as those derived from differential evolution principles [33], to ensure the proper optimization is achieved and derived in an acceptable lengths of time.

These simulations involved two risk factors and one outcome. However, we recognize that this scenario is seldom observed in real epidemiologic practice, in which more typically there are multiple covariates that may predict the outcome. Nevertheless, the simulations illustrated how SL techniques can potentially improve upon common methods currently applied in epidemiologic research when nonlinearities are present. The linear separation produced by the LR model was exemplified with a low-dimensional problem that contained all of the features of higher dimensional problems. The kernel mapping transformed the original relationship to a feature space where linear techniques are applicable without assuming interaction forms, although a valid kernel must be determined.

## Conclusions

The work demonstrated the potential benefits derived from applying SL techniques to nonlinear epidemiologic type problems. Integrating SL techniques with epidemiologic research may aid researchers in defining complex exposure/disease relationships. These applications will require validation in population-based studies and further rigorous comparisons with existing methods.

## Authors' contributions

JJH and WHL developed the statistical learning analysis methods. All authors contributed equally in the manuscript conception, experimental design, and composition. All authors read and approved the final manuscript.
